# Identification of a novel EphB4 phosphodegron regulated by the autocrine IGFII/IR^A^ axis in malignant mesothelioma

**DOI:** 10.1038/s41388-019-0854-y

**Published:** 2019-07-03

**Authors:** Pierluigi Scalia, Giuseppe Pandini, Vincenzo Carnevale, Antonio Giordano, Stephen J. Williams

**Affiliations:** 1 Sbarro Institute for Cancer Research and Molecular Medicine and Center for Biotechnology, Biology Department, Temple University, Philadelphia, PA 19122 USA; 2 Istituto Somatogene Per la Ricerca Onco-Genomica, ISOPROG, Caltanissetta, 93100 Italy; 3 Somatolink Research Foundation, Philadelphia, PA 19102 USA; 4 Endocrinology, Department of Clinical and Experimental Medicine, Garibaldi-Nesima Medical Center, University of Catania, 95122 Catania, Italy; 5 Institute of Computational Molecular Science, College of Science and Technology, Temple University, Philadelphia, PA 19122 USA; 6 Department of Medical Biotechnology, University of Siena, Siena, 53100 Italy

**Keywords:** Diagnostic markers, Mesothelioma

## Abstract

Malignant mesothelioma is a deadly disease with limited therapeutic options. EphB4 is an oncogenic tyrosine kinase receptor expressed in malignant mesothelioma as well as in a variety of cancers. It is involved in tumor microenvironment mediating angiogenesis and invasive cellular effects via both EphrinB2 ligand-dependent and independent mechanisms. The molecular network underlying EphB4 oncogenic effects is still unclear. Here we show that EphB4 expression in malignant mesothelioma cells is markedly decreased upon neutralization of cancer-secreted IGF-II. In particular, we demonstrate that EphB4 protein expression in malignant mesothelioma cells depend upon a degradation rescue mechanism controlled by the autocrine IGF-II-insulin receptor-A specific signaling axis. We show that the regulation of EphB4 expression is linked to a competing post-translational modification of its carboxy-terminal tail via phosphorylation of its tyrosine 987 by the Insulin receptor isoform-A kinase-associated activity in response to the autocrine IGF-II stimuli. Neutralization of this autocrine-induced EphB4-phosphorylation by IGF-II associates with the increased ubiquitination of EphB4 carboxy-terminal tail and with its rapid degradation. We also describe a novel Ubiquitin binding motif in the targeted region as part of the identified EphB4 phosphodegron and provide 3D modeling data supporting a possible model for the acute EphB4 PTM-driven regulation by IGF-II. Altogether, these findings disclose a novel molecular mechanism for the maintenance of EphB4-expression in malignant mesothelioma cells and other IGF-II-secreting cancers (IGF2omas).

## Introduction

EphB4 is a venous endothelial developmental marker and a membrane tyrosine kinase interacting with EphrinB2 which is expressed in arterial endothelial cells during vasculogenesis [1–3]. EphB4 has been found overexpressed in malignant cancer cells where it has been associated to a variety of cancer-promoting effects, including morphogenesis, angiogenesis, invasion, and metastasis [4–8] and its pharmacologic block or kinase activity deficiency counteracts such effects [9, 10]. EphB4 it is thought to exert its physiologic functions via extracellular binding with EphrinB2, a receptor kinase itself which is able to stimulate EphB4 tyrosine kinase activity and trigger both a forward and reverse signal. Although EphB4 forward signal, induced as ligand for the ephrinB2 kinase receptor expressed in contiguous cells, has been shown to involve PI3K [11], Crk [12], and Rho [13] not much is known about the mechanisms that regulate its reverse signaling effects during angiogenesis, migration and invasion. At present, a number of evidences, as for the paradoxical switch in biologic effects (from promoting to suppressing) in certain cancers [14], as well as Eph/Ephrins ecto-domain shedding which causes post-translational truncation of the receptor kinases [15] all point at the possibility that EphB4 reverse cellular effects in the cells where it is expressed may be the result of both EphrinB2 ligand-dependent and ligand-independent signals. Although EphB4 expression has been found upregulated in response to IGF-I in vitro [5], the IGF-II autocrine loop has been shown to be a hallmark for a variety of cancers in vivo where its systemic effects are responsible for the cancer-associated syndrome previously known as non-insulin-dependent-hypoglycemia (NIDH) later referred as to IGF2omas [16]. The role of IGF-II in development [17] and cancer has been widely described [18–26]. Notably, in a transgenic mouse model developing pancreatic cancer, IGF-II expression was instrumental at the switch between benign versus malignant proliferation, marked by significant increase in the cancer highly vascular phenotype [20]. IGF-II was also identified as the most overexpressed gene in human angiomas by large expression screening of more than 10,000 genes [27]. Nonetheless, IGF-II’s cellular effects and the specific receptor and signaling mediators in supporting angiogenic, invasive and metastatic cellular behaviors have been poorly elucidated. The findings reported herein identify EphB4 as a specific IGF-II target via an insulin receptor isoform A- triggered signal and are in line with a strict functional dependence of EphB4 from the autocrine IGF-II-IR^A^-stimuli in order to modulate its reverse signaling and the cellular effects resulting by their co-expression in cancer.

## Results

### EphB4 is co-expressed with the autocrine IGF-II ligand and kinase receptors in malignant cancer cell lines

**Fig. 1:**
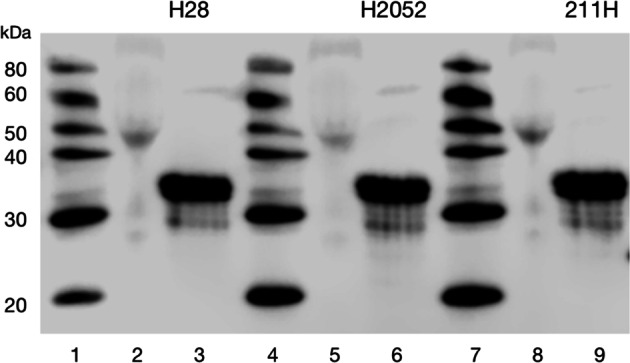
IGF-II is found secreted as high molecular weight variants in conditioned media of Mesothelioma cell lines. Secreted IGF-II from conditioned media collected in NCI-H28, NCI-H2052, and NCI-MSTO-211H was detected by immunoprecipitation. The immunocaptured ligand at the end of a neutralization block treatment was collected in prot-A agarose beads, denatured and used for western blotting on a semi-native 15% PAGE. Lanes 1, 4, 7: Genscript markers, lanes 2, 5, 8: conditioned Media control (1/10 input), lanes 3, 6, 9: immunocaptured IGF-II from mesothelioma cell lines conditioned media

In order to test whether EphB4 and the autocrine IGF-II system were part of a common mechanism in cancer, we profiled a number of cancer cell lines for the expression of the IGF-II loop autocrine components combining both known data with experimental validation (Table 1 and Fig. 1). Semi-native PAGE analysis of immunoprecipitated IGF-II from conditioned media of NCI-H28, NCI-H2052, and NCI-MSTO-211H malignant mesothelioma cell lines provided a homogeneous immune-reactive band with an apparent MW of 34KDa (Fig. 1). This is consistent with previously described high molecular weight (HMW) variants originating through combined retention of the IGF-II pre-pro-peptide domain E and its cell-specific O-glycosylation [28]. These HMW variants have been shown to bear the capability to bind and activate the IR^A^ and the IGFIR while showing less affinity for other IGF-II binding proteins [29]. EphB4 expression fully overlapped with the IGF-II secretion status along with the expression of the two IGF-II signal transducing tyrosine kinase receptors (IR^A^ and IGF1R) in all the overtly aggressive cancer cell lines we tested including mesothelioma, breast carcinoma, prostate carcinoma, sarcoma, human hepatoma and colon carcinoma (Table 1) but in normal immortalized cells (MCF-10A).

**Table 1:** EphB4 is co-expressed with the autocrine IGF-II loop components in malignant cancer cells and EphB4 levels decrease upon autocrine IGF-II stimuli neutralization in cultured cells

IGF-ll Autocrine components					EphB4 mRNA/ protein	EphB4 protein downregulation by IGFII mAb	References
Cancer Cell line	IGF-II mRNA/ protein	Secreted IGF-II	IGF1R mRNA/ protein	IR^A^ (A/B ratio %)			
*Mesothelioma* MST0211H NCI-H2052 NCI-28H NCI-H2373	+	+/+++	**+**	55–100%		+ (54–82% inhib.)	present study
*Breast ca* MCF7 MDA-MB468 T47D BT20 MDA-MB157	+	+ / + + +	+ /–	50–95%	+ *	t.b.d.	Sciacca et al. 1999 *Present study
*Breast* *(immortalized)* MCF-10A	negative	n.d.	+	38%	n.d.*	t.b.d.	Sciacca et al. 2002 *Present study
*Prostate ca* PC3, DU145 LNCaP	+	+ / + + +	+	50–100%	+ *	t.b.d.	Pandini (unpublished data) Kimura et al. 1996 *Present study
*Sarcoma* SAGS-2 SKUT1 Sj-RH30	+	+	+ /–	96–100%	+ *	t.b.d.	Sciacca et al. 2002 *Present study
*Liver ca* HepG2	+	+	+	50–80%	+*	t.b.d.	Pandini et al. 2002 Greenhall 2018 *Present study
*Colon ca* HT29 HTC116 SW480 ARO.1	+	+/+++	+	50–100%	+*	t.b.d.	Vella (personal communication) Yang et al. 2011 *Present study

Levels of protein and/or gene transcripts for IGF2, IGF1R, Insulin receptor and EphB4 along with secreted IGF-II ligand were tested in a number of cancer cell lines. The relative decrease in EphB4 protein levels upon secreted IGF-II neutralization in mesothelioma cell lines is also shown. Relative decrease of immunoblot EphB4 bands upon IGF-II mAb-mediated neutralization was quantitated and percentage of control inhibition reported. Results summarized in the table are representative of three or more independent experiments

### EphB4 protein expression strictly relies upon the autocrine IGF-II steady-state signal in malignant mesothelioma cell lines

**Fig. 2:**
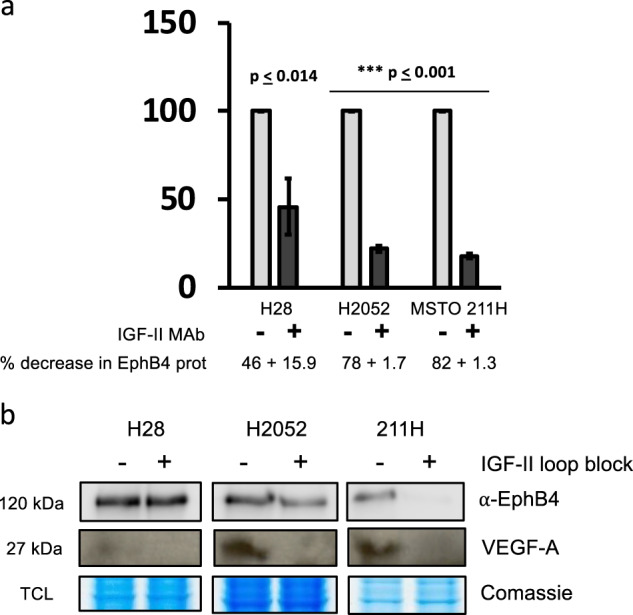
EphB4 and VEGF-A levels depend upon intact IGF-II autocrine loop in Mesothelioma cell lines. **a** Column graph of EphB4 levels in mesothelioma cell lines conditionally pre-treated with a neutralizing anti-human IGF-II Ab. EphB4 densitometric values were normalized to the Coomassie staining of TCL and data represented as mean with sem of three experiments. Statistical analysis was performed using two-tailed Student’s *t*-test. **b** Immunoblot of EphB4 (upper row), VEGF-A (mid row) and pan-Actin (bottom row). Total cell lysates (TCL, VEGF-A, and pan-Actin) or immunoprecipitates (EphB4) from mesothelioma cancer cell lines conditionally pre-treated with a neutralizing anti-human IGF-II Ab were used

**Fig. 3:**
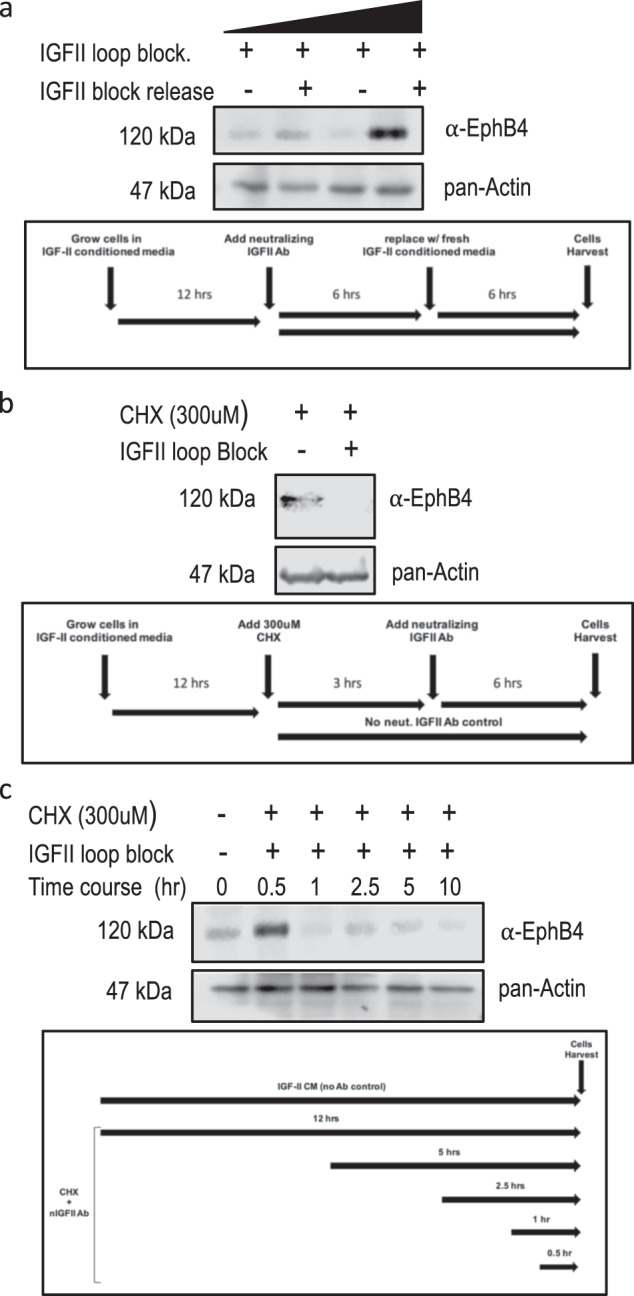
The IGF-II autocrine signal is required to insure EphB4 steady-state expression and protein stability in cancer cells. **a** Reversibility of the antibody-induced autocrine IGF-II block in MSTO 211H. This was evaluated with Ab-induced neutralization of cancer cell-secreted IGF-II followed by release for additional 12 h with fresh conditioned media (without neutralizing Ab) from the same cell line (lanes 2, 4). 50 µg and 200 µg were used for EphB4 immunoprecipitation. **b** Dependence of the autocrine IGF-II rescue effect on de novo EphB4 synthesized protein by pre-treatment with Cycloheximide for 6 h (left) followed by Ab-neutralization of secreted IGF-II (right). **c** Time course effect of CHX and IGF-II neutralizing antibody on EphB4 protein levels (without CHX pre-treatment). In each experiment, 15 µl aliquot of the of pre**-**normalized TCL were used for pan-actin internal control (western blot). The experiments shown are representative of three independent experiments. The experimental workflow used in **a**, **b** and **c** is shown

In order to verify whether the observed co-expression trend in EphB4 and autocrine IGF-II in cancer cell lines was functionally correlated, we set up an experiment using three IGF-II secreting malignant mesothelioma cell lines (NCI-H28, NCI-H2052, and MSTO-211H). We then used a neutralizing IGF-II antibody to modulate its autocrine effects in vitro. As summarized by the effect displayed in Fig. 2, the neutralizing IGF-II antibody treatment was able to inhibit EphB4 protein expression between 54 and 82 percent among the malignant mesothelioma cell lines studies (Fig. 2a, b upper row). The antibody-mediated block of the IGF-II autocrine stimuli decreased the levels of VEGF-A in the same cell lines (Fig. 2b, middle row). This is consistent with other published literature showing a double dependence between the VEGF system and the IGF system [30], the pre-normalized protein levels among parallel tested conditions were further confirmed by Coomassie stain (Fig. 2b, bottom row). In order to study the nature and reversibility of this effect we set up three types of experiments in MSTO211H. First, we looked at the reversibility of the observed autocrine block by replacing the neutralizing antibody with plain fresh conditioned media and looked at the EphB4 endogenous levels following 12 h from the block release (see experimental workflow under each Fig. 3). The result of this approach (Fig. 3a) confirmed that EphB4 levels in these cells strictly depends upon an intact autocrine IGF-II stimuli and that this mechanism is fully reversible. Next, we studied the dependence of the observed effect on de novo synthesized EphB4 by using Cycloheximide (CHX) and looked at its effects on the EphB4 regulation by autocrine IGF-II. The results of this approach are shown in Fig. 3b, c. The pre-treatment with CHX findings confirmed the hypothesis that autocrine IGF-II regulates de-novo synthesized EphB4 protein expression and therefore it has to be considered a post-transcriptional effect. Time course treatment with CHX on IGF-II deprived MSTO211H (Fig. 3c) further confirmed the observed trend (see 0 versus 10 h treatment) in spite of an observed CHX-induced transient increase of the EphB4 steady-state levels at 30 min post treatment before the expected drop.

### EphB4 is a tyrosine phosphorylation target of autocrine IGF-II via IR^A^ but not the IGFIR at tyrosine 987, in its C-terminal tail end

**Fig. 4:**
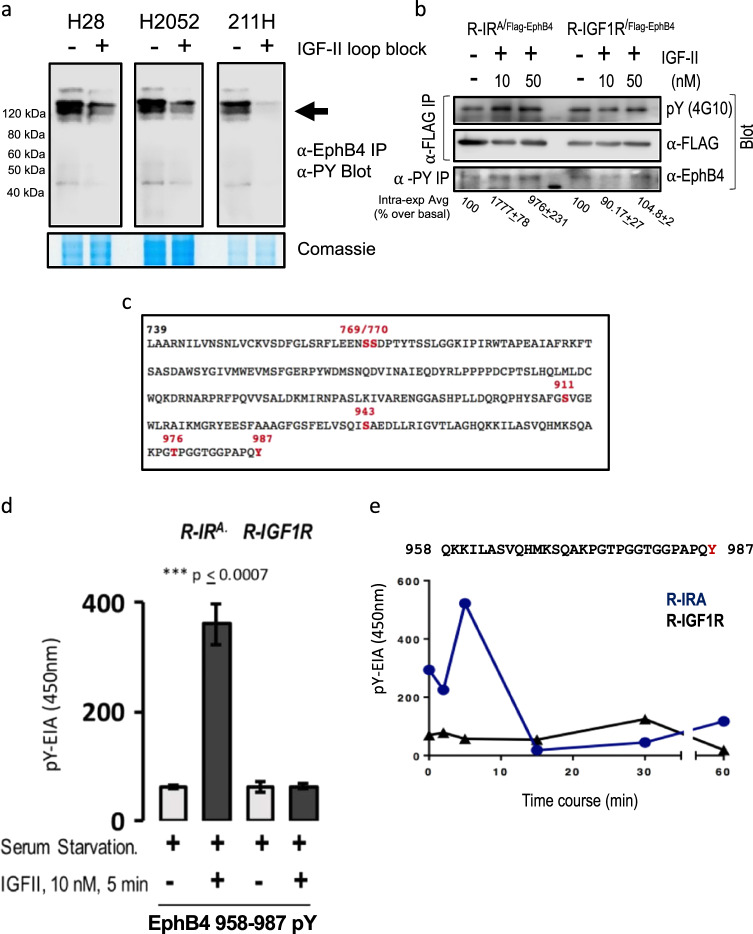
EphB4 is a tyrosine phosphorylative target of IGF-II via activation of the Insulin Receptor isoform A on its Tyr987 residue. **a** Effect of the autocrine IGF-II signal neutralization on the EphB4 tyrosine phosphorylative status in H28, H2052, and MSTO211H mesothelioma cell lines. Tyrosine phosphorylation was detected by immunoblot in anti-EphB4 immunoprecipitates (upper). The input used for IP was obtained by Coomassie stain (bottom). **b** R- (MEFs from igf1r null mice) stably expressing either the hIR^A^ or the hIGF1R were transiently transfected with hEphB4 (N-Flag), serum starved for 12 h and stimulated with human synthetic IGF-II at either 10 or 50 nM for 3 min before cell harvesting, solubilization, protein normalization, and anti-Flag (M2) beads or anti-phosphotyrosine immune precipitation. An aliquot (1/10^th^) of the IP input was used for Flag prot expression control. Intra-experimental average of increased Tyr phosphorylated EphB4 over unstimulated condition is shown. **c** Known in vivo phosphorylated residues in human EphB4 intracellular region (739–789) sequence obtained from Uniprot (Swiss prot) database showing tyrosine 987 as the only tyrosine residue phosphorylated in vivo in the EphB4 intracellular portion via large phospho-proteomic analysis. **d** In vitro kinase assay (by EIA) on EphB4 958–987 synthetic substrate using IGF-II pre-stimulated R-hIR^A^ and R-hIGF1R MEF cell lysates after either 5 min stimulation with 10 nM IGF-II post overnight serum starvation. **e** Time course of human IGF-II stimulation in post-serum starved R-IR^A^ and R-IGF1R MEF cells at 5, 15, 30, and 60 min post-stimulation. The experiments shown are a representative out of three independent experiments. The experiments shown in **a** are representative of three independent experiments (mean plus SE). Statistical analysis was performed using two-tailed Student’s *t*-test

To shed further light on the biochemical nature of the autocrine IGF-II-dependent expression of EphB4, we looked at possible post-transcriptional mechanisms and targeted modifications induced by IGF-II on EphB4 that might justify this dramatic protein decrease upon neutralization of the IGF-II autocrine signal. EphB4 had been previously found to be one of the phospho-tyrosine targets of IGF-II but not of insulin via the insulin receptor-A [31]. Therefore, we looked at the tyrosine activation of autocrine IGF-II on immunoprecipitated EphB4 in mesothelioma cancer cell lines either in absence or presence of neutralizing IGF-II antibody block. The result observed in mesothelioma cell lines (Fig. 4a) showed that antibody-mediated inhibition is able to decrease tyrosine phosphorylation on EphB4 immunoprecipitates at inhibition levels higher than those exerted on the whole protein (compare Fig. 2b with Fig. 4a) suggesting that autocrine IGF-II could affect EphB4 tyrosine phosphorylation independently or preceding the reduction on steady-state protein expression. Therefore, we set up experiments using engineered mouse embryo cell lines carrying a null mutation for the igf1 receptor and stably expressing either the insulin receptor A, exon 11 minus or the human IGFI receptor, R-IR^A^ and R-IGFR [19], where we transiently co-expressed a full length N-terminal Flag-tagged human EphB4 construct. We then acutely stimulated (3 min) these cell lines following overnight serum starvation using both a non-supra-physiological concentration of human synthetic IGF-II (10 nM) as well as a supra-physiological concentration (50 nM).

As shown in Fig. 4b, EphB4 tyrosine phosphorylation was clearly increased in response to IGF-II via the IR^A^. Unexpectedly, we observed no significant increase in the serum starved (basal) status in R-IGF1R cells using different approaches (compare upper and lower rows in Fig. 4b). Next, in order to pinpoint specific tyrosine residues responsible for the observed effect, we conducted a search on the UniProt Knowledge Base [32] seeking for possible tyrosine residue(s) targetable by IGF-II on human EphB4 in its intracellular region (spanning region 739–987 of the protein). The search is summarized in Fig. 4c [33, 34]. Interestingly, we found that the last C-terminus residue of EphB4 corresponding to tyrosine 987 is, so far, the only tyrosine found phosphorylated in vivo by large scale phospho-proteomic screening [35]. Not less interesting was the finding that among eukaryotic EphB4 orthologs only rodent EphB4 is missing of Y987 (Fig. 6b) making it an ideal model where to express and study human EphB4 as we chose to do. In order to test whether the Y987 residue identified by UniProt Knowledge Base search was indeed the specific target for IGF-II/IR^A^-dependent Tyrosine phosphorylation we set up an in vitro kinase assay using a synthetic peptide spanning region 958 to 987 of human EphB4 as a potential in vitro substrate for IGF-II pre-stimulated cellular extracts from either R-IR^A^ or R-IGF1R cells. We chose this peptidic stretch because it is located downstream to the previously described SAM domain [33] and it showed a number of potentially relevant post-translational modification (PTM) sites previously found modified in vivo. The result shown in Fig. 4d, confirmed that the Tyrosine responsible for the effect displayed in Fig. 4b is indeed Tyrosine 987 and confirmed the observation that the Insulin Receptor A (IR^A^) but not the IGF1R mediates such effect. Further time course for the same in vitro approach (Fig. 4e) showed a peak for EphB4 tyrosine 987 phosphorylation at 5 minutes post-IGF-II stimulation.

### EpHB4 is targeted by ubiquitination in cancer cells and its ubiquitination levels are inversely related to the autocrine IGF-II-induced Tyr987 phosphorylation

**Fig. 5:**
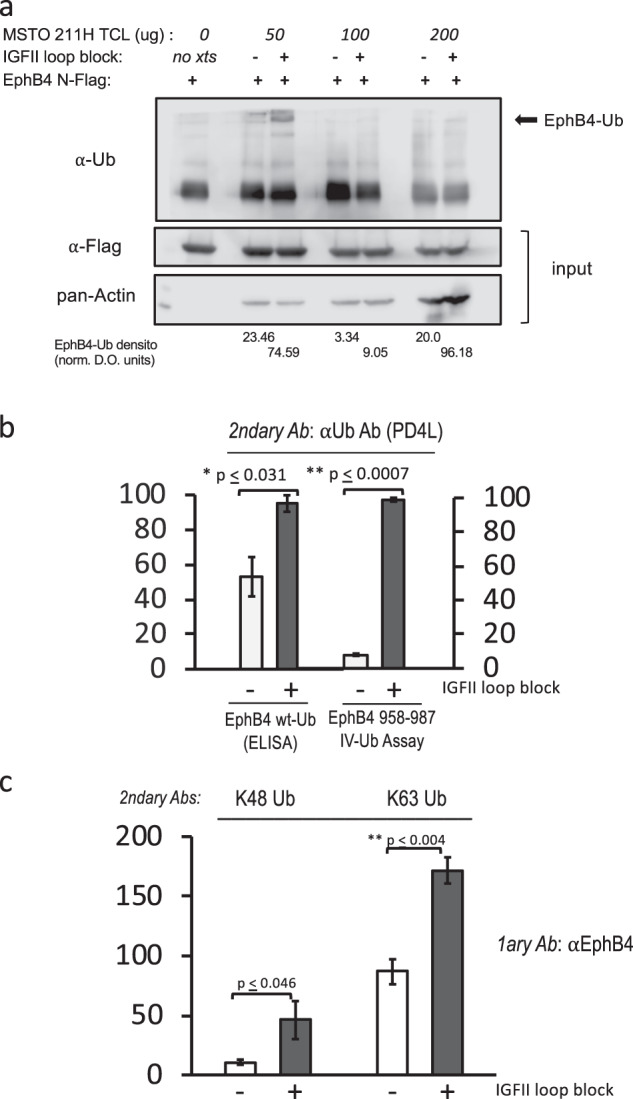
EphB4 is regulated by ubiquitination and the ubiquitination of its carboxy terminus region is prevented by the IGF-II autocrine stimuli. **a** In vitro ubiquitination of immobilized N-Flag hEphB4 from transiently expressed R-IR^A^ by MSTO211H cell extracts with or without ex-vivo block of the autocrine IGF-II stimuli (upper row); 1/5th of the immobilized Flag-EphB4 used as substrate for in vitro ubiquitination was used as reaction input control (middle row). The amounts of MSTO211H total cell extracts (50–200 µg) used as source of native ubiquitin ligases in the assay are shown as pan-actin levels (bottom row). The relative rate of ubiquitination based upon densitometric evaluation of the specific bands in the upper row is reported. **b** Total ubiquitination of endogenous EphB4 immuno-captured from MSTO211H (ELISA, left columns) and the corresponding ubiquitination of immobilized EphB4 958–987 peptide (EIA, right columns) by the same extracts both in presence or absence of autocrine IGF-II block. **c** Effect of autocrine IGFII block on K48 and K63 Ubiquitin branched proteins in EphB4 immunoprecipitates from MSTO211H. The experiments shown are representative of three independent experiments (mean plus SE for **b** and **c** are shown). Statistical analysis for ELISA and EIA experiments in **b** and **c** was performed using two-tailed Student’s *t*-test

The demonstration that EphB4 Y987 phosphorylation is a target of autocrine IGF-II-IR^A^ signaling axis prompted us to look at other PTMs possibly linked to the observed IGF-II-induced degradation rescue effect. Therefore, we focused on ubiquitination and looked at the possibility that EphB4 could be a ubiquitination target with respect to the IGF-II autocrine stimuli status. In order to do so, we used a number of approaches specifically meant to clarify the relationship between EphB4 tyrosine phosphorylation and its ubiquitination (Fig. 5). We first set up an in vitro ubiquitination assay by using N-Flag tagged human EphB4 as a putative substrate which was transiently expressed and immunocaptured from R-IR^A^ cells and then performed an in vitro assay using MSTO211H cell extracts with or without ex-vivo neutralization of secreted IGF-II as source of unknown ubiquitin ligases. To minimize the possible effect of previous post-translational modifications on hEphB4 expressed in R-IR^A^ cells used as substrate, the N-Flag EphB4 recovered in M2 (anti-flag) beads was first dephosphorylated using CIAP and then washed with high stringency buffer to remove possible bound proteins before adding MSTO211H cell extracts (see under methods). As shown in Fig. 5a, this approach clearly indicated that EphB4 is indeed a target of ubiquitination and that this effect is induced by Ub-ligases in cells deprived of the IGF-II self-stimulatory signal. This finding demonstrates the existence of an inverse relationship between the IGF-II autocrine-induced phosphorylation of Tyrosine 987 and EphB4 ubiquitination. Under the assay conditions minimal auto-ubiquitination activity was also detected (see No extracts condition in Fig. 5a). Interestingly, we constantly observed higher in vitro ubiquitination levels using 50 micrograms cell extracts from IGF-II signal-deprived MSTO211H in the assay suggesting a limiting effect in the enzyme/ substrate ratios between EphB4 substrate vs E1–3 native ligases under the condition tested. Nonetheless, normalization of the highly ubiquitinated EphB4 band measured with densitometry by the amount of reaction substrate (EphB4 N-flag) and cell extracts, respectively, consistently provided a 2.7–4.8 fold ubiquitination increase when cells were deprived of the IGF-II autocrine stimuli (Fig. 5a). The observed inverse trend between the IGFII self-stimulation status and EphB4 ubiquitination observed in the in vitro assay was also shown for the endogenous/native EphB4 pool from the same cancer cells using ELISA (Fig. 5b, left panel). Even more specific was the finding that the last carboxy-terminal 30 amino acids of hEphB4 were able to reproduce the ubiquitination trend observed with full length EphB4 when ubiquitination was assayed in vitro by an enzymatic immuno-assay we developed using EphB4 C-terminus peptide 958–987 as a substrate against MSTO211H extracts (Fig. 5b, compare right with left panel). This last finding specifically supports a scenario where most of the IGF-II-dependent ubiquitination residues are hosted by the C-terminal EphB4 domain spanning aa 958–973. The type of Lysine ubiquitination, namely on Lys48 versus Lys63, has been linked to degradative versus regulatory properties of the protein targeted by ubiquitination as reviewed in ref. [34]. Therefore, we also tested the role of these types of ubiquitination in the immuno-captured EphB4 complex from MSTO211H cell extracts either with or without block of the autocrine IGFII stimuli. As shown in Fig. 5c, both Ub-K48 and Ub-K63 appear to play a role in EphB4 protein regulation by IGF-II. K63 was the most prominent type of ubiquitination associated to EphB4 in the cell model studied. In all cases, ubiquitination was clearly increased upon block of the IGF-II autocrine stimuli confirming the inversely related trend between ubiquitination versus its Tyr987 phosphorylation.

### Identification of a ubiquitin binding-like motif in EphB4 958–974 C-terminus region and comparative phylogenetic analysis of EphB4 C-terminus

**Fig. 6:**
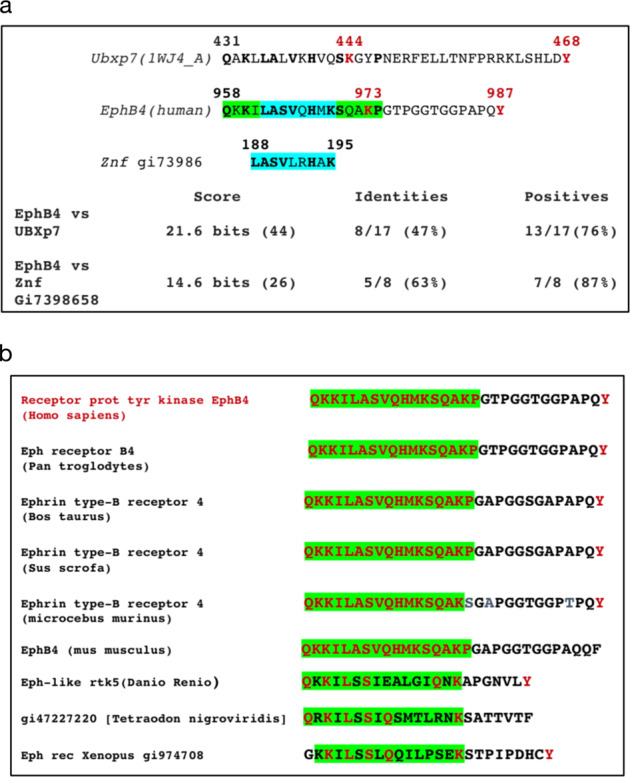
Identification of a novel Ubiquitin-binding motif on the EphB4 C-terminal region and phylogenetic comparison of C-terminal domain in EphB4. **a** identification of a combined Ubiquitin-like (green) and a Znf-protein-related (light blue) motif in the EphB4 C-terminus domain (958–974). **b** Phylogenetic comparison of EphB4 C-Terminus phosphodegron. Green: ubiquitin-like consensus motifs. Red: human sequence conserved residues. Note the C-terminal tyrosine conservation throughout species but in rodents

Due to the counteracting ubiquitination/phosphorylation PTM trend observed on EphB4 958–987 C-terminal region, downstream to the last known EphB4 functional domain (SAM) which has been found involved in EphB4 dimerization [33] we performed a conserved-domain search comparison for known UBX and UBL protein domains followed by blast alignment against the above human EphB4 intracellular carboxy-terminal region. Surprisingly, one of these domains, carried by UBX-domain-containing protein 7 (UBXbP7) corresponding to position 431–438 of its C-terminus protein sequence (ref 1WJ4_A) shared 47% amino acid identity and a 76% positive score with the EphB4 region spanning aa 958–974 which is contained within the last 30 amino acid stretch of the EphB4 carboxy terminus ending with the tyrosine residue in position 987 (Fig. 6a, green background, upper row). Further analysis of this 17 aa motif for known other Ubiquitin like and ubiquitin-binding domains allowed us to identify an unstructured znf inter-domain motif of 7aa encompassing the aa 963–970 of hEphB4 with 63% identity and 87% positive score (protein ID, znf7398658) as shown Fig. 6a bottom row (light blue background). Worth noticing, the identified motif contains Lysine 973 which has been found ubiquitinated in vivo [35]. Furthermore, the conserved UBX/znf-like motif is located immediately downstream to Threonine 976 which has also been found phosphorylated in vivo [32, 35]. These findings provide a molecular basis and possible dynamic scenarios for the growth-factor-induced regulatory effects underlying the observed phosphorylative/ubiquitination switch. A further phylogenetic comparison of the immediate upstream region to the tyrosine 987 in human EphB4 was conducted via pBlast using EphB4 protein sequences from both mammalian and non-mammalian orthologues with regards to the identified UBX-like motif and at the light of the observed inverted pattern of tyrosine phosphorylation versus ubiquitination in this specific aa stretch that might narrow down our interest in potential mechanisms regulated by Tyrosine 987 phosphorylation or its phylogenetic counterpart. The result of this search is shown in Fig. 6b with consensus for the UBX-like motif shown in green background and Tyrosine 987 and its counterpart in EphB4 orthologues shown in red. The implications of such finding are included under Discussion. Interestingly, this shared motif in Ubxp7 is located immediately upstream to a C-terminus tyrosine residue also in this protein suggesting that this newly identified consensus region might have a preserved function possibly related to the control of protein interaction depending upon the phosphotyrosine activation status and the conditional docking and/or ubiquitination of this UBX/znf-like motif. Such features entitles EphB4 region 958–987 as a putative novel Phosphodegron [36].

### 3D dynamic modeling of human EphB4 Carboxy-terminal domain upon Y987 phosphorylation

**Fig. 7:**
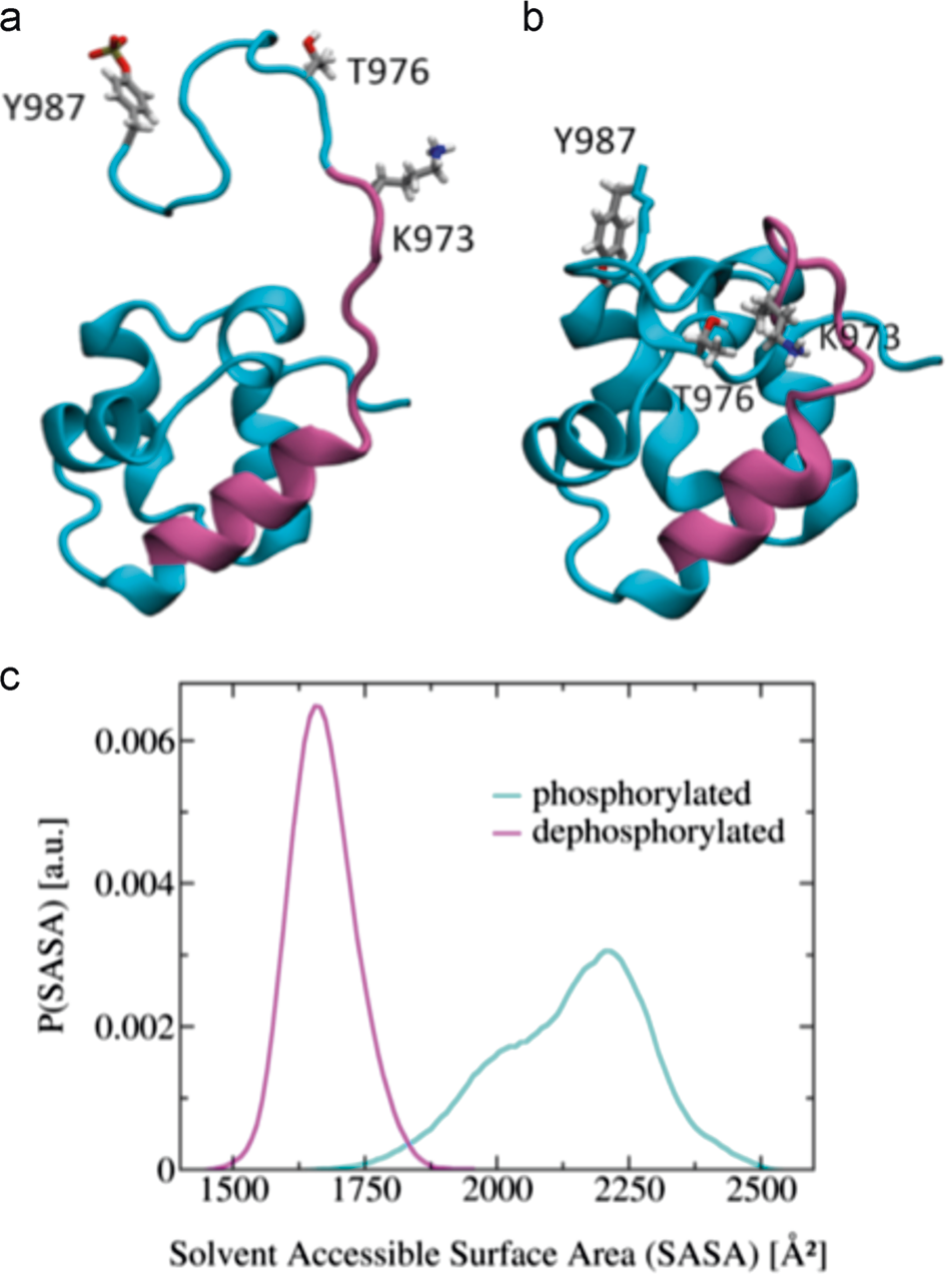
Effect of Tyr987 phosphorylation on EphB4 Carboxy-Terminal 3D conformation and UBX-Znf-Like binding motif access measured as solvent availability by dynamic modeling. Structural consequences of Y987 phosphorylation. **a**, **b** Cartoon structure of the SAM domain with the putative UBX-Znf-Like binding domain (amino acids 958 through 974) highlighted in purple. The side chains of the amino acids K973, T976, and Y987 are shown as sticks. The structural models in **a**, **b** correspond to the phosphorylated and dephosphorylated state of Y987, respectively. **c** Solvent accessible surface area (SASA) of the C-terminus loop (amino acids 970 through 987). The SASA was calculated for each frame of the molecular dynamics trajectory; the resulting distributions are shown for the phosphorylated and dephosphorylated cases in cyan and purple, respectively. Note that Y987 phosphorylation causes a statistically significant increase of the SASA

To investigate the structural rearrangement resulting from phosphorylation of tyrosine 987 by the IGF-II/IR^A^ signal, we performed molecular dynamics simulations on structural models of the SAM domain along with the full C-terminal loop (see Fig. 7). To this end, we used the structure determined via X-ray crystallography, pdb code 2QKQ, as a starting configuration, after including the C-terminus loop amino acid sequence (not detected in the experimental electron density). We initialized two distinct models in which Y987 is either phosphorylated or dephosphorylated. In neither of the two simulations the loop showed formation of secondary structure. In both cases, this region remained unstructured and relatively dynamic. However, we observed a major structural difference in the relative arrangement between the loop and the rest of the SAM domain. In the dephosphorylated case, the loop bound tightly to the globular domain with almost all the side chains of the loop engaged in interactions with the rest of the protein. By contrast, in the Y987 phosphorylated case, the loop was detached from the globular domain and was mostly observed in an extended conformation (Fig. 7a, b). As a result, the side chains are, on average, exposed to the solvent and accessible to other cellular interaction partners. In particular, upon phosphorylation, the solvent accessible surface area increased approximately from 1690 to 2250 Å^2^, i.e. more than 30%. The consequences of this 3D modeling study are discussed in detail under the homonymous section.

## Discussion

The present study discloses a novel molecular network functional to the maintenance of malignant features in cancer cells. This mechanism is triggered by the post-translational modifications induced by the autocrine IGF-II signal targeting the angiogenic/oncogenic membrane receptor kinase EphB4. EphB4 belongs to the family of EphB/Ephrin membrane tyrosine kinases [37, 38]. Its ability to establish both a forward and a reverse cellular signaling during blood vessel formation and cardiovascular development, upon extracellular binding to its cognate ligand/receptor partner EphrinB2 has been elegantly demonstrated in genetically-modified mice [1, 3]. The demonstration of EphB4 cancer-promoting effects in a variety of malignant cancer types [5, 13, 39] has provided the rational for targeting EphB4 as a mean to block its reverse signaling in the therapeutic setting [9, 40, 41]. However, EphB4 has been also associated with a paradoxical tumor-suppressing effect in an immortalized non fully malignant cancer cell (MCF-10A) [14]. It is possible that the functional uncoupling of the EphrinB2-EphB4 extracellular interaction, such as that caused by ectodomain shedding as well as any other molecular mechanism leading to functional uncoupling of the EphrinB2- EphB4 interaction [7, 14, 42, 43], might account for the contextual switch from differentiative (e.g., morphogenic and vasculogenic) to undifferentiated phenotype (namely, disorganized migration, and primitive blood vessel formation) in solid tumors. A possible scenario for the EphrinB2-independent EphB4 cancer-promoting effects come from the observation that EphB4 is able to bind and eventually trans-activate other membrane TK receptorial systems such as the EGFR [44]. Although the EphB4 cancer-promoting effects have been established, little is known about the exact mechanisms by which EphB4 exerts such effects. The present study clearly establishes EphB4 as a controlled downstream target for the autocrine IGF-II loop which, per se, is a hallmark for the majority of human and mammalian solid cancers [16, 18, 20, 26] and demonstrate that EphB4 protein expression in mesothelioma cell lines is strictly dependent upon a steady-state interplay between IGF-II induced tyrosine phosphorylation and the ubiquitination of its C-terminal region acting as a molecular switch for EphB4 protein stabilization versus degradation. The fact that EphB4 IGF-II-dependent phosphorylation modifications observed in this study precede the drop in de novo synthesized protein levels provide a required cause/effect association between the observed phosphorylation changes and the decrease in EphB4 steady-state levels. We propose such mechanism as a key checkpoint towards gaining/maintaining a malignant phenotype in certain cancers. Although a proteomic study has previously shown that EphB4 is an IGF-II (but not insulin) specific tyrosine phosphorylative target of the Insulin receptor-A [31], the specific tyrosine residue and the functional role of such phosphorylation was unknown. The present study identifies EphB4 intracellular last residue, Tyrosine 987, as the specific target of the autocrine IGF-II signal in cancer and it links its phosphorylation status with the protein stability and overall lifespan of EphB4 expression. The fact that the EphB4 last carboxy-terminal 30 amino acids were sufficient to recapitulate the phosphorylation and ubiquitination pattern of the full length molecule further points at the structural independence of this carboxy-terminal region of EphB4 in mediating the stabilization/degradative switch function in response to the IGFII-IRA stimuli. Such type of alternative phosphorylative/ubiquitination switch has been indeed shown for a number of highly regulated cellular proteins and the protein regions responsible for the phosphorylative control of ubiquitination and the associated degradation have been coined as “phosphodegrons” [36]. A review of the known in vivo modified amino acid residues on the EphB4 intracellular portion via UniProt search [32, 35] suggests that Lys973 could play a role in the observed ubiquitination/degradation trend due to its proximity to Tyr987 as part of a novel phosphodegron spanning the last 30 c-terminal amino acids of EphB4. Interestingly, using a conserved domain database search we found this Lysine to reside within a UBX-like motif having a core similarity with a sequence found in a Znf protein module. This further suggests that the described IGF-II dependent phosphorylation may have a role in the interplay between EphB4 and the binding with unknown Ubiquitin-regulated factors at this region. Our study also shows that the autocrine IGF-II-IR^A^ signal-deprived EphB4 is associated with an increase in both K48- and K63-branched ubiquitin chains. These Ub-branched variants have been involved in ubiquitin-mediated degradation and protein unfolding processes, respectively [34, 45]. Surprisingly, the EphB4 carboxy-terminal tridimensional features obtained via dynamic modeling apparently conflicted with the expected spatial configuration. In fact, the Tyr987 dephosphorylated form, which rapidly degrades was found to bear a more compact structure than the respective phosphorylated version as also suggested by solvent accessibility. The opposite increased accessibility of Lys973 and the UBX/Znf-like motif suggested by the molecular dynamic analysis upon phosphorylation of Tyr987 associating with a decrease in ubiquitination and degradation of the EphB4 protein could be explained by the consensual phosphorylation of Threonine 976 as a permissive event following phosphorylation of Tyrosine 987 towards the molecular regulation of EphB4 protein by autocrine IGF-II. According to this model, supported by the dynamic modeling results, a second IGF-II regulated (threonine) kinase would be required upon increased accessibility of Thr976 triggered by Tyr987 phosphorylation. Threonine 976 has indeed also been described to be an in-vivo phosphorylated residue in EphB4 [35] and is located two residues downstream Lys973. In this local context, phosphorylation of Thr976, due to the additional negative charge and the ensuing repulsive interaction, would prevent ubiquitin priming of EphB4 Lys973 by specific ubiquitin-binding factors. The net result would justify the observed increased lifetime in expressed EphB4 protein and the maintenance of the underlying oncogenic signals. Indeed, our observation that IGF-II antibody-mediated block of its autocrine stimuli induced a dramatic reduction of the overall EphB4 protein in presence of serum and (other) growth factors-rich cultural/microenvironmental conditions is compatible with IGF-II being the sole growth factor responsible with the phosphorylation of the EphB4 C-terminal 30 aa tail. At present the proposed model for a Tyr987/Thr976 double phosphorylation by IGF-II towards the regulation of the described EphB4 bona-fide phosphodegron, while fully fitting with the present findings, will requires further experimental confirmatory work. It is worth noticing that the EphB4 C-terminal aa stretch described herein partially overlapped (16 amino acids) with the C-term side of the predicted 70aa-long EphB4 SAM domain sequence (NCBI conserved domain ID: 2QKQ_A [33]. This further raises the possibility that the autocrine IGF-II mechanism shown in here, besides regulating the steady-state expression levels of EphB4, might also affect EphB4 dimerization in vivo. Additional structure/function studies will clarify the exact role of the EphB4 C-terminal tail with specific regards to the identified phospho-degron. The mechanism described by this study also supports a scenario where the signal triggered by interaction of IGF-II and the Insulin receptor short isoform (IR^A^, exon 11 minus) differs from the one mediated solely via the IGFR in the fact that the observed phosphorylation of EphB4 by autocrine IGF-II appears to be linked solely to the IR^A^ but not the IGFIR kinase activity. Indeed, the IGFIR had been also shown to upregulate EphB4 in prostate cancer [5] but under the conditions used, such effect could be linked to both transcriptional and post-transcriptional mechanisms such as the presence of Ins receptor/IGFIR hybrids [46]. Our findings support that the IGF-II-IGFIR signal does not play a role in the control of EphB4 protein stability in cancer. A major confirmation about the underscored role of the IGF-II-IR^A^ axis in cancer has come from the failure of the IGF-IR blockers developed in the last few decades in phase-II clinical trials when used as monotherapy [47] suggesting that anti-cancer strategies blocking the IGF-II-IR^A^ signal along with that generated by the IGFII-IGFIR axis cannot be disregarded in vivo. The concept of a differential and diversified role of the Insulin receptor and the IGF1R towards controlling growth, aging- and cancer-related effects also comes from the recent observation that the IR but not the IGF1R are under the phylogenetic control of a specific ubiquitin-linked degradative system regulating its steady-state protein level in C.Elegans [48]. At last, our finding that VEGF-A levels were reduced in parallel with EphB4 under the cultural cell conditions used raises the question of an interdependence between Hypoxia (common in solid cancers and known HIF and VEGF triggering factor) and the observed IGF-II/IRA-EphB4 regulatory axis under the same conditions. Indeed, IGF-II has been shown to upregulate VEGF-A in HCC and such effect is synergistic with hypoxia [30]. Furthermore, EphB4 has also been found upregulated by Hypoxia in mouse skin [49]. Under our experimental conditions, hypoxia was shown by a conditioned media measured pH of 6.9 under which both IGF-II and EphB4 could have been potentially upregulated by HIF. However, under such cultural conditions (hypoxic, serum-containing and growth-factor rich), the neutralization of secreted IGF-II was able to significantly downregulate EphB4 as well as VEGF-A suggesting a major role for IGF-II as a mediator of HIF effects in malignant mesothelioma cells. Such question will require further attention in the future for its possible biologic and actionable implications. All together, these observations provide the rational for foreseeable studies aimed at the identification of the molecular components responsible for the observed regulation of the EphB4 phosphodegron by the IGFII/IRA stimuli towards causing EphB4 over-expression in cancer. Ultimately, our study showing the dependency between the autocrine IGF-II -generated stimuli and the steady-state EphB4 protein expression in cancer suggests an underscored role for this novel degradation rescue mechanism as a potential player in the tumorigenic switch [50] in malignant mesothelioma.

## Materials and methods

Source of reagents and antibodies and the working concentrations used in this study, can be found in Table 2.

**Table 2:** List of antibodies and related reagents

Antibodies and reagents				
Antigen (/Ab Clone)	Animal source/clonality	Working concentration	Provider	Clone/product#
IGF-II	goat monoclonal	1.15ug/ml	R&D Systems	AF292
Insulin Receptor (MA-20)	Mouse monoclonal	1:2000	Santa Cruz	MA-20/sc57344
EphB4 (H-10)	mouse monoclonal	1:2000	Santa Cruz	H-10/sc-365510
EphB4 (5G2F8)	mouse monoclonal	1:2000	Santa Cruz	5G2F8/sc130081
Ubiquitin (P4D1)	mouse monoclonal	1:1000	Santa Cruz	P4D1/sc8017
Ubiquitin K-48 (HRP) (D9D5)	rabbit monoclonal	1:1000	Cell Signaling	D9D5/12805s
Ubiquitin-K63 (HRP) (D7A11)	rabbit monoclonal	1:1000	Cell Signaling	D7A11/12930s
Phospho-Tyr (4G10)	mouse monoclonal	1:10000	UBI/Millipore	4G10/16–103
Phospho-Tyr (4G10)-HRP	mouse monoclonal	1:10000	EMD Millipore	4G10/16–316
Flag M2-agarose beads	mouse monoclonal	2 µl/ml	Sigma Aldrich	A2220
Normal rabbit IgG	rabbit polyclonal	1 mg/ml	Sigma Aldrich	I5006–10MG
Anti-mouse IgG-HRP	chicken monoclonal	1:2000	Santa Cruz	sc2954
Anti-IgGk-LC-HRP	Mouse monoclonal	1:1000	Santa Cruz	sc156102
Anti-mouse-IgG-mag beads (Dynabeads)	Goat	2 µl/ml	Dynal Biotech	110.33
Streptavidin mag beads	n/a	2 µl/ml	Genscript	L00424
Protein A/G Plus beads	n/a	10ul/condition	Santa Cruz	Sc-2003
Ubiquitin, human synthetic	n/a	800 ng/rxn	Boston Biochemical	U-100H
Cycloheximide	n/a	300 µM	Millipore Sigma	C7698

### Expression constructs and synthetic peptides

The Human EphB4 N-Flag expression construct was purchased from Sino Biological (Wayne, PA). EphB4 955–987 N-Biotin was obtained by New England Peptides Inc. (Boston, MA); EphB4 955–987 Y- > L was obtained by Genscript (Piscataway, NJ).

### Cell treatments and exogenous recombinant/tagged proteins expression

MSTO211H were obtained from Dr. Steven Albelda (University of Pennsylvania) and NCI-H28 and NCI-2052H were obtained through the Fox Chase Cancer Center cell culture facility and all underwent confirmatory STR genotypization. R-IR^A^ and R+ mIgf1r null mouse embryo fibroblasts (MEFs) constitutively expressing, respectively, the human IR isoform A and human IGF1R were previously described [19]. Cancer cell lines were maintained in RPMI, 10% Fetal Bovine Serum (SIGMA) while MEFs were cultured in DMEM, high glucose, 10% FBS under 0.3 µg/ml Puromycin selection. Cationic lipid transfections of MEFs were carried out in OPTIMEM (Gibco-Life Technologies) optimized media using Lipofectamine 2000 reagent (Thermo Scientific, Invitrogen, Carlsbad, CA) according to the manufacturer recommendations. Secreted IGF-II conditioned media was obtained by collecting the culture media of the selected IGF-II secreting cancer cell lines at a minimum of 85% confluency. Autocrine IGF-II loop neutralization was obtained by adding a neutralizing anti-human IGF-II antibody (R&D systems, clone AF292) to the conditioned culture media of IGFII secreting cancer cells and the neutralization effect evaluated following 12 h treatment. Conditioned media least measured pH value observed at the end of each treatment was 6.9 with no morphological changes between treated and untreated conditions (not shown). Following treatment the CM media was collected for IGF-II immunoprecipitation, PAGE (15%) under semi-native conditions and western blot while cells were harvested and lysed on ice for whole cell extracts preparation as described below. Protein amounts were measured using an aliquot of the lysates using BCA reagent (SIGMA, Saint Louis, MO) and lysates used for protein experiments were brought to the same the concentration using ice-cold lysis buffer with full protease/phosphatase inhibitors cocktail used throughout the study (see complete recipe below). IGFII stimulation of MEFs was carried out following 12 h serum starvation in serum-free media followed by replacement of serum-free media with fresh starvation media containing 10 nM human IGF-II (Sigma-Aldrich, St. Louis, MI) and 0.1% BSA for the indicated times followed by cell harvesting. Transient transfection of MEFs with N-Flag EphB4 expression vector was obtained by cationic lipid-mediated transfection (Lipofectamine 2000) in OptiMEM low growth factor media according to the manufacturer recommendations. Whole cell extracts used in this study were obtained with NP40 containing lysis buffer (1% NP40, NaCl 137 mM, 20 mM Tris 7.4, 1 mM MgCl, 1 mM CaCl_2_, 10% Glycerol) containing protease/phosphatase inhibitors (1mM DTT, 2 mM Benzamide, 10 mM b-glycerol Phosphate, 10 mM NaF, 2 mM NaVO_3_, 10 µg/ml Pepstatin, 10 µg/ml Leupeptin, 10 µg/ml Aprotinin, 100 nM Okadaic Acid, 0.5 mM PMSF). Tagged proteins expression was evaluated by western blot using the antibodies listed herein. Immunoprecipitation of N-Flag tagged, Full-length EphB4 from transiently transfected cell lysate was performed at 4 °C for 2 h in gentle rotation using M2-N-Flag agarose beads from SIGMA. All other immunoprecipitations were performed similarly but using Agarose A/G beads (Santa Cruz) preceded by 30 min preclearing of the lysates using a non-specific antibody (rabbit serum, SIGMA).

### Western blotting

Western blotting was performed as previously described [25] post-protein transfer on PVDF membranes (Hibond, GE-Amersham, Pittsburgh, PA). Non specific blocking was performed with 3% Bovine Serum Albumin in PBST. Primary antibodies (see reagents list) were used at the specified concentration/dilution ranging from 1:1000 to 1:2000 in PBST. HRP-conjugated antibodies (species-specific, Rockland) were used at 1:2000 dilution. The chemiluminescence signal was generated with the “Supersignal West Fempto” substrate (Thermo-Scientific, Waltham MA) and detected using a digital gel detection system Odyssey Fc (Li-Cor Bioscience, Lincoln, NE).

### In vitro protein tyrosine kinase assay

EphB4 987 tyrosine phosphorylation was confirmed by Enzymatic Immuno-Assay by coating a 96 well plate (Nunc Maxisorb) with 2 µg/well EphB4 synthetic peptide corresponding to its region 958–987 (Genscript) as under the ELISA protocol. The in vitro phospho-tyrosine reaction in presence of native whole cell extracts (40 µg) was carried out in 50 mM Hepes pH 7.5, 150 mM NaCl, 2 mM MnCl_2_, 10 mM MgCl, 30 mM ATP, 100 µM GTP, 0.1% NP40 and 0.05% bovine serum albumin in presence of a complete protease/ phosphatase inhibitors’ cocktail for 2 h at 22 °C. The interfering effect of Tyr-phosphorylated proteins bound to the peptide following in vitro kinase reaction was eliminated by incubating 15 min in high salt buffer (1 M KCl, 1% deoxycholate 20 mM Tris, pH 8.0, 0.5 mM EDTA) at 4 °C followed by TBST washes. Tyrosine phosphorylation of EphB4 Y987 was detected by incubating the post-reaction coated peptide with anti-phosphotyrosine monoclonal antibody (4G10, Pierce) HRP-conjugated at 0.3 µg/ml in 50 mM Hepes pH 7.5, 150 mM NaCl, 0.05% Tween20, 1% bovine serum albumin with protease/phosphatase inhibitors. Four TBST washes were performed following each step. The HRP signal was further amplified using the ELAST Elisa Amplification System (Perkin Elmer, Waltham MA) according to the manufacturer conditions. A quantitative colorimetric reaction was carried out by adding TMB substrate (Thermo-Scientific, Waltham, MA) and the resulting signal detected after stopping the colorimetric reaction with 2 M sulphuric acid by optical read at 450 nM fixed wavelength on a spectrophotometer (uQuant, BIOTEK). The specificity of the detected signal for EphB4 Y987 phosphorylation (the only tyrosine present in the synthetic peptide) was further strengthened by performing a parallel in vitro reaction using a synthetic peptide bearing a Y987- > L single residue replacement and considering any visible signal as the non-specific background.

### Ubiquitination studies

#### In vitro Ub assay of Flag-EphB4, full length (western blot detection)

Full length human EphB4 transiently expressed in R-IR^A^ was pulled down from 125 µg cell extracts using anti-Flag (M2) agarose beads and used as a ubiquitination target in vitro by MSTO211H cancer cell extracts (50–200 µg) as a source of native E1–3 ligases in presence of purified human Ubiquitin (800 ng), 20 mM Tris-HCl pH 7.5, 5 mM MgCl_2_, 2 mM ATP, 1 mM DTT, along with the appropriate protease inhibitors. Extracts-driven ubiquitination of immobilized N-Flag-EphB4 was carried out for 1.5 h at 37 °C. Reaction stopped by PBS washing. The flagged ubiquitination target released from immune-beads by denaturation at 94 °C in gel loading buffer containing 50 mM DTT. The supernatant resolved on a 12% polyacrylamide SDS-gel and proteins transferred on PVDF membrane. Ubiquitination status of EphB4 revealed by western blotting using a monoclonal anti-Ubiquitin antibody (clone P4D1) as described above.

#### EphB4 Ubiquitination by ELISA

The EphB4 immune-capture antibodies (Santa Cruz) used for coating (0.3 µg/ml) was adsorbed onto 96 well plates (Maxisorb™, Nunc Millipore Sigma) in H_2_CO_3_/HCO_3_- buffer, pH 9.6 o/n at 4 °C followed by non-site blocking in 20 mM Tris pH 7.4, 150 mM NaCl, 0.05% Tween20, 1% bovine serum albumin at 56 °C for 30 min. 10–40 µg cell lysates were incubated for 2 h at 4 °C in presence of protease/phosphatase inhibitors. Primary antibodies incubation was carried out in 50 mM Hepes pH 7.5, 150 mM NaCl, 0.05% Tween20, 1% Bovine Serum Albumin with protease/phosphatase inhibitors incubating for 2 h at 22 °C. Primary monoclonal antibodies raised against either total ubiquitin (Santa Cruz, P4D1 clone, Dallas TX) at 1:200 dilution or K-48 and K-63 branched ubiquitin chains (Cell Signaling, Danvers MA) at 1:100 dilution were used. The ELAST Elisa Amplification System (Perkin Elmer, Waltham MA) was used depending upon the resulting signal intensity. Either OPD (Millipore Sigma, St. Louis MO) or TMB (Thermo Scientific, Waltham, MA) were ELAST Elisa Amplification System (Perkin Elmer, Waltham MA) used as colorimetric substrates for ELISA and reactions ended by adding 2 M sulphuric acid. Optic Density was quantified by spectrophotometric reading at 492 (OPD) or 450 (TMB) wavelengths in a BIOTEK reader (uQuant). All conditions were carried out in triplicates.

#### In vitro Ub assay of EphB4 958–987 (by EIA)

EIA using EphB4 958–987 synthetic peptide as plate-coated substrate was also adopted to confirm ubiquitination by native E-ligases from MSTO211H cell extracts to the last EphB4 C-terminal 30 amino acid residues. EphB4 synthetic peptide (2 µg) was used to coat each individual well out of a 96 well plate in order to set up triplicate reactions. The in vitro Ub reaction was performed as described above for the full length ubiquitination study. Ubiquitin detection (using clone P4D1) and all following steps were identical to the ELISA protocol using up to the detection level.

### RT-PCR

PCR for IRA/B, IGF1R, IGF2, and EphB4 was performed from cDNA of a variety of IGF-II secreting or control IGF-II negative cancer cell lines as previously described [18, 39]. Relative quantification to a co-expressed housekeeping gene (beta-actin) was performed using densitometry. For mesothelioma cell lines total RNA was obtained from freshly cultured cell lines using TRIZOL™ reagent (Thermo-Scientific, Waltham, MA) and Direct-zol™ RNA Miniprep Plus column purification kit (Zymo Research, Irvine CA). cDNA was obtained by using the ReadyScript® cDNA Synthesis Mix (Millipore Sigma, Burlington MA) according to the manufacturer instructions on an ABI thermocycler and PCR performed using Accuprime Taq Polymerase (Thermo-Scientific, Waltham, MA) and optimized buffer at 94 °C denaturation (30 s), 55 °C annealing (30 s) and 72 °C extension (45 s) sequential thermocycle for 35 cycles. Primers sequence used for PCR were: EphB4 F: 5’ TCC TGC AAG GAG ACC TTC AC 3′, EphB4 R: 5′ GAG AGG CCT CGC AAC TAC AT 3′, IRA/B F: 5′ AAC CAG AGT GAG TAT GAG GAT 3′, IRA/B R: 5’ CCG TTC CAG AGC GAA GTG CTT 3′, IGFIR F: 5′ ATG AAG TCT GGC TCC GGA 3′, IGFIR R:: 5′ CTC ATG TTG ATG GCG ACG 3′, IGF2 F: 5′ GAA GTC GAT GCT GGT GCT C 3′, IGF2R: 5′ CCT CCG ATT GCT GGC CAT CT 3′. RT-PCR results were combined with protein data published elsewhere [18, 51–53] towards the assembly of Table 1.

### 3D dynamic modeling study

Molecular dynamics trajectories were collected for the SAM domain with and without a phosphate bound to Y987. The simulations were performed in implicit solvent (Generalized Born Surface Area) using GROMACS-5.1.5 (www.gromacs.org, Uppsala University, Sweden) and the amber99sb forcefield [54]. Simulations were performed at constant temperature (300 K) using a velocity rescaling scheme [55]. The time step for integration of the equations of motions was set to 1 fs. The overall length of the molecular dynamics trajectory for each state was 100 ns.

### Bioinformatic analysis

Analysis of EphB4 958–987 protein sequence for functional domains/motifs and phylogenetic comparison of EphB4 c-terminal was performed using the NCBI tools (protein db, conserved domain db, and pBlast protein seq comparison tool). The search of in vivo modified residues on the same region was performed via Swissprot/UniProt DB and phosphosite.org open source databases.

### Statistical analysis

The statistical analysis was performed using a two-tailed unpaired Student *t*-test following *F*-test for variance equivalence using Microsoft Excel (Redmond, CA) and GraphPad Prism Software (La Jolla, CA). A *p* < 0.05 was considered as significant. Results are expressed as mean + standard error of the mean and are representative of at least two or three independent experiments in triplicate.
